# Parenteral organophosphorus poisoning in a rural emergency department: a case report

**DOI:** 10.1186/1756-0500-6-524

**Published:** 2013-12-09

**Authors:** Gyanendra Malla, Bibhusan Basnet, Rais Vohra, Shyam Prasad Lohani, Ajay Yadav, Vivek Dhungana

**Affiliations:** 1 Department of General practice and Emergency Medicine, B. P. Koirala Institute of Health Sciences, Dharan, Nepal; 2 Department of Emergency Medicine, UCSF Fresno Medical Center, Fresno, CA, USA; 3 Nepal Drug and Poison Information Centre, Kathmandu, Nepal; 4 Department of Emergency Medicine, Kathmandu Medical College, Kathmandu, Nepal

**Keywords:** Chlorpyriphos, Cellulitis, Organophosphorus, Parenteral, Poisoning

## Abstract

**Background:**

Poisoning is a common presentation in the emergency department. Oral exposures to organophosphorus compounds are especially frequent in rural and agricultural regions of South Asia and throughout the developing world.

**Case presentation:**

Here we report a case of deliberate self-harm with an organophosphorus pesticide via the relatively uncommon parenteral route. A young woman injected herself with chlorpyriphos. Although the cholinergic effects were mild, cellulitis and abscess development were noted as a result.

**Conclusion:**

Resource limited agricultural countries like Nepal present health care workers with numerous challenges in poisoning management. This case represents a rare but potentially morbid method of agrochemical poison exposure.

## Background

Organophosphorus (OP) poisoning is common wherever agriculture is a common profession [1]. It has been a major public health problem in developing countries [2]. The usual routes of OP poisoning are oral, inhalation or trans-cutaneous absorption; both unintentional and intentional poisoning. Other routes of exposure are uncommonly reported. Here we present a case of a young woman who intentionally inflicted self-harm by injecting herself with chlorpyriphos, which is a moderately toxic OP compound [3].

## Case presentation

An eighteen-year-old woman was brought to the Emergency Department (ED) of a tertiary level hospital in a largely agricultural region of Nepal with an alleged history of chlorpyriphos poisoning. She had injected herself with the compound. At the time of presentation, it was uncertain whether the patient gave herself an intravenous, subcutaneous, or intramuscular dose of chlorpyriphos. However, as a professional paramedic it is likely that she was attempting intravenous administration since she had the expertise and access to syringes and needles.

The exposure reportedly occurred 24 hours prior to presentation, during an attempted suicide. She had presented with a few episodes of vomiting and lacrimation at the rural hospital. Her initial management for the poisoning was done with atropine and pralidoxime (2-PAM). Nasogastric suction and Foley catheterisation was done at the rural district hospital. Intravenous access was secured. She was then referred for higher level of intensive care to a tertiary care centre.

On presentation to our hospital she was alert, well oriented, and cooperative, with a Glasgow coma score of 15. Her vital signs were stable (Pulse: 76 beats per minute, BP: 110/70 mmHg, RR: 14 breaths per minute, Temperature: 99°F, SPO_2_: 98% on room air). Pupils were of normal size and reactive to light bilaterally. Plantar reflexes and deep tendon reflexes were normal bilaterally. There were no fasciculations. Examination of the chest showed bilateral equal air entry with no crepitation or wheezing. As she had minimal features of OP poisoning, atropine was not continued as a treatment.

**Figure 1:**
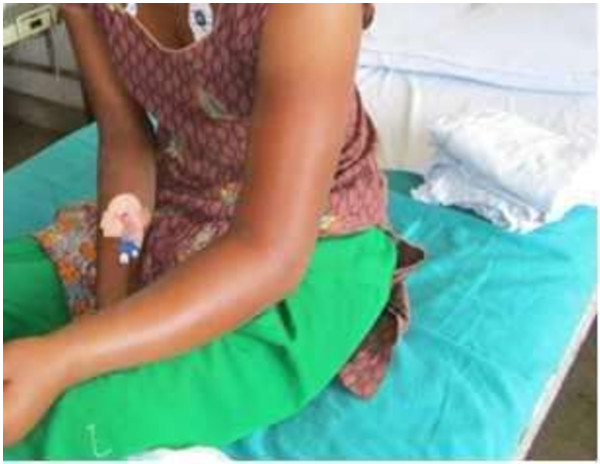
Patient at the emergency department with a swollen, erythematous left arm after the parenteral organophosphorous exposure.

Investigations at admission showed normal renal function, liver function, and normal serum levels of sodium, potassium, calcium, and magnesium. Arterial blood gas analysis showed a pH of 7.40, PO_2_: 95 mmHg and PCO_2_ of 37 mmHg. Her cholinesterase levels were not assayed due to unavailability of the test at our facility. After 12 hours of symptom-free interval, she developed swelling at the OP injection site (Figure 1). Her total leukocyte count was measured, which resulted as 26,400/cu mm, with 77% neutrophils and 23% lymphocytes. The injection site was warm to touch and erythematous, but without fluctuance. Radiographic analysis with an ultrasound was done to rule out an abscess, foreign bodies, or subcutaneous air. Even though there was no fever, a diagnosis of cellulitis, combined with irritant dermatitis due to the hydrocarbon component of the OP solvents, was suspected. The patient was given intravenous antibiotics antibiotics to cover a broad spectrum of microbial agents (cloxacillin and metronidazole) as well as oral Ibuprofen for pain.

After seven days of treatment, the patient developed worsening swelling and increased pain. Her motor function as measured via the Medical Research Council (MRC) scale on the involved hand was 3/5 in comparison to the uninvolved contralateral limb (4/5). She also developed fever recorded up to 101°F. Her daily total leucocyte counts had no decreasing trends despite antibiotic treatment, which triggered evaluation for an abscess at the injection site. Ultrasound-guided aspiration of the lesion was done, and 5 mL of pus was aspirated followed by incision and debridement. Her clinical course after that was uneventful. She was discharged after 12 days of hospitalization.

Following her clinical recovery, she was evaluated by psychiatrists and it was revealed that injection of poison was an impulsive act due to familial disputes. Follow up visit was planned after 1 week of daily dressing changes and oral antibiotics at home.

## Discussion

OP compounds, also known more generally as cholinesterase inhibitors, are widely used compounds in the form of pesticides, therapeutic agents, and nerve agents in chemical warfare. Exposure to OPs can be accidental or related to suicidal gesture. Acute exposure to OPs can lead to death or serious illness [4]. Poisonings are particularly common in rural areas and developing countries where even the most potent agents are widely available [5]. Chronic exposure is also possible, particularly among poor rural populations where men, women, and children all work and live in close proximity to fields and orchards where chemicals are applied and stored [1].

OP compounds are cholinesterase inhibitors, which result in excess acetylcholine in autonomic ganglia and terminal muscarinic synapses. Since these agents affect both nicotinic and muscarinic receptors, a wide variety of symptoms and signs can be seen (Table 1) [4].

**Table 1:** Symptoms and signs of organophosphorus poisoning

**Muscarinic receptors**	**Nicotinic receptors**	**Central receptors**
**Cardiovascular**	**Cardiovascular**	**General effects**
Bradycardia	Tachycardia	Anxiety
Hypotension	Hypertension	Restlessness
**Respiratory**	**Musculoskeletal**	Ataxia
Rhinorrhoea	Weakness	Convulsions
Bronchorrhoea	Fasciculations	Insomnia
Bronchospasm	Cramps	Dysarthria
Cough	Paralysis	Tremors
**Gastrointestinal**			Coma
Nausea/vomiting			Absent reflexes
Increased salivation			Respiratory depression
Abdominal cramps			Circulatory collapse
Diarrhoea			
Faecal incontinence			
**Genitourinary**			
Urinary continence			
**Eyes**			
Blurred vision			
Increased lacrimation			
Miosis			
**Glands**			
Excessive salivation			

Clinical presentations of OP poisoning can be broadly classified as secondary to the (a) muscarinic effects, (b) nicotinic effects, and (c) central receptor stimulation [3]. Clinically, the features of OP poising can be sub-classified into three stages: (i) acute cholinergic crisis, which manifests within 24 to 72 hours due to accumulation of acetylcholine at muscarinic and nicotinic sites and accumulation within the central nervous system (CNS) resulting in seizures and altered sensorium; (ii) intermediate syndrome, which manifests after 24 to 96 hours as a precipitous weakness of ocular, neck, limb, and respiratory muscles; and (iii) a delayed, often permanent peripheral neuropathy can result from the toxicity of certain OP compounds [6].

Organic phosphates are co-formulated with potent hydrocarbon solvents that can act as local irritants. Cellulitis and local tissue damage can thus result from cutaneous or subcutaneous OP exposures, although this is uncommon compared to the cholinergic and neurotoxic sequalae described above. A case report by Volk *et al*. documents typical cholinergic symptoms and rhabdomyolysis as a late manifestation of parenteral OP exposure, possibly due to an irritant mechanism [7]. Prior case reports by Pandit and Raina describe intravenous and intramuscular routes of OP exposure, presenting with cholinergic symptoms and later with complications of cellulitis [8-10].

A case of OP poisoning can be a great challenge for the ED team in resource-constrained settings. Initial workup for management is based on the history of exposure and the presence of characteristic clinical features mentioned above. Laboratory confirmation for OP poisoning consists of red blood cell (RBC) or plasma cholinesterase levels. But these tests are expensive and not universally available in low middle income countries (LMIC's) like Nepal. Rajapakse et al. have recently reported the use of a field-based kit (Test-mate ChE 400) for rapid cholinesterase level activity [11]. In addition, tests directed to identify metabolic derangements (such as electrolytes, glucose, Blood Urea Nitrogen, creatinine, liver transaminases, arterial blood gases or oximetry, Electrocardiography monitoring, and chest x-ray) should also be performed. Additionally, diagnostic workup for parenteral OP poisoining may require a variety of approaches, including plain radiographs, computerized tomography, and/or ultrasonographic studies around the swollen/erythematous areas, to identify for signs of cellulitis, necrotizing fasciitis, retained needles, and/or impending abscess.

Clinical management of OP poisoning centers primarily on treatment of bronchial secretions, which can overwhelm the respiratory function of the patient and lead to a death which is often characterized as “drowning on dry land.” To combat this process, it is imperative to give the antimuscarininc agent atropine, frequently in large doses. An initial dose of 1.2 mg should be doubled every 5 minutes, as atropine takes only a few minutes to work, and reassessment of the patient every 5–10 minutes is necessary in order to determine the level at which the patient’s secretions have been controlled and respiratory distress has abated. The use of pulse-oximetry and pulmonary auscultation are the most reliable methods to assess the patient. Pupillary dilation and tachycardia are expected side effects of high-dose atropine; these should not be used as endpoints in initial management. More problematically, excess atropine can cause agitation, confusion, and hyperthermia from loss of sweating functions.

These challenges are regularly faced by junior clinicians working in emergency wards, and experienced staff should be present to help resuscitate and stabilize the severely poisoned patients. Recent guidelines for antidote use have been published by Eddleston *et al.*[12]. In addition to atropine, the cholinesterase regenerating agent pralidoxime (initial and maintenance doses) is recommended to reverse nicotinic signs of OP poisoning (especially fasciculations and respiratory muscle weakness), but pralidoxime must be initiated before the cholinesterase enzyme has irreversibly bound to the organophosphate agent. This process is known as “aging” of the enzyme and pralidoxime is not useful after aging occurs. The use of pralidoxime has been increasingly controversial due to the relatively new data calling its efficacy into question and more research is needed to identify which patients will most benefit from this antidote [13]. Lastly, the use of benzodiazepines is warranted to combat seizures (due to CNS cholinergic excess) and control atropine-related agitation.

The patient in this case report did not have severe cholinergic toxicity from the OP exposure, but she did develop infectious complications from the unique route of self-harm she chose. Indeed, cellulitis and abscess formation is commonly described when recreational drugs such as heroin and amphetamine are injected [14,15]. Bacterial contamination of the needles and improper sterile technique contribute to infectious complications, which can also include more serious sequelae such as endocarditis and necrotizing fasciitis.

Finally, it is important to recognize that hydrocarbons (such as those present in OP formulations used as solvents) can be highly irritating to body tissues. When hydrocarbons are accidentally injected by paint guns into the digits or hands, a widespread tenosynovitis or chemically induced necrotizing fasciitis can occur, because these chemicals are able to travel rapidly along fascial planes far from the site of injection. Soft tissue damage due to abscess, cellulitis, necrotizing fasciitis and tenosynovitis can be detected with ultrasound imaging, which should be used early and frequently to assess the patient’s severity of insult and response to treatments. Anti-inflammatory, analgesic and antibacterial medications were used to treat the secondary bacterial infection, and abscess formation ultimately required local aspiration and incisional drainage. The initial choice of antibacterial agent should cover skin flora such as Staphylococcus/Streptococcus species, and further treatment can be guided by blood and tissue culture sensitivity patterns.

## Conclusion

This case represents a relatively rare method of OP administration and a unique set of local complications subsequent to injection. We conclude that, while there was a limited cholinergic syndrome from this subcutaneous exposure to chlorpyriphos, the combination of contaminated needle and the OP solvent formulation contributed to the complication of cellulitis and delayed abscess in this patient. Resource limited agricultural countries like Nepal present health care workers with numerous challenges in poisoning management. Clinical signs and available diagnostic tests such as ultrasound should be used to assess the severity and nature of any local complications from parenteral poisoning exposures.

## Consent

Written informed consent was obtained from the patient for publication of this Case Report and any accompanying images. A copy of the written consent is available for review by the Editor-in-Chief of this journal.

## Abbreviations

ED: Emergency Department; OP: Organophosphorus; CNS: Central nervous system; MRC: Medical Research Council; RBC: Red blood cell.

## Competing interests

The authors declare that they have no competing interests.

## Authors’ contributions

GM, BB, AY handled the case in the ED. GM, BB, RV, SPL conceived the case report, and participated in its design. BB, RV, SPL and VD drafted the manuscript and sequence alignment of the report. BB, RV, GM and VD reviewed the literature. All authors read and approved the final manuscript.
